# The impact of *MTHFR* 677C → T risk knowledge on changes in folate intake: findings from the Food4Me study

**DOI:** 10.1186/s12263-016-0539-x

**Published:** 2016-09-29

**Authors:** Clare B. O’Donovan, Marianne C. Walsh, Hannah Forster, Clara Woolhead, Carlos Celis-Morales, Rosalind Fallaize, Anna L. Macready, Cyril F. M. Marsaux, Santiago Navas-Carretero, Rodrigo San-Cristobal, Silvia Kolossa, Christina Mavrogianni, Christina P. Lambrinou, George Moschonis, Magdalena Godlewska, Agnieszka Surwillo, Jildau Bouwman, Keith Grimaldi, Iwona Traczyk, Christian A. Drevon, Hannelore Daniel, Yannis Manios, J. Alfredo Martinez, Wim H. M. Saris, Julie A. Lovegrove, John C. Mathers, Michael J. Gibney, Lorraine Brennan, Eileen R. Gibney

**Affiliations:** 1 Institute of Food & Health, University College Dublin, Dublin 4, Ireland; 2 Human Nutrition Research Centre, Institute of Cellular Medicine, Newcastle University, Newcastle, NE4 5PL UK; 3 Hugh Sinclair Unit of Human Nutrition and Institute for Cardiovascular and Metabolic Health, University of Reading, Reading, RG6 6AR UK; 4 Department of Human Biology, NUTRIM, Maastricht University, Maastricht, The Netherlands; 5 Department of Nutrition, Food Science and Physiology, University of Navarra, Pamplona, Spain; 6 CIBERobn, Fisiopatología de la Obesidad y Nutrición, INstituto de Salud Carlos III, Madrid, Spain; 7 IDISNA, Instituto de Investigación Sanitaria de Navarra, Pamplona, Spain; 8 ZIEL Research Center of Nutrition and Food Sciences, Biochemistry Unit, Technische Universität München, Munich, Germany; 9 Department of Nutrition and Dietetics, Harokopio University, Athens, Greece; 10 National Food & Nutrition Institute, Warsaw, Poland; 11 TNO, Microbiology and Systems Biology Group, Zeist, The Netherlands; 12 Eurogenetica Ltd, Salisbury Road, Burnham-on-Sea, TA8 1HX UK; 13 Department of Human Nutrition, Faculty of Health Sciences, Medical University of Warsaw, Warsaw, Poland; 14 Department of Nutrition, Institute of Basic Medical Sciences, University of Oslo, Oslo, Norway

**Keywords:** *MTHFR*, Methylenetetrahydrofolate reductase 677C → T genotype, Genetic risk knowledge, Folate, Personalised nutrition

## Abstract

**Background:**

It is hypothesised that individuals with knowledge of their genetic risk are more likely to make health-promoting dietary and lifestyle changes. The present study aims to test this hypothesis using data from the Food4Me study. This was a 6-month Internet-based randomised controlled trial conducted across seven centres in Europe where individuals received either general healthy eating advice or varying levels of personalised nutrition advice. Participants who received genotype-based personalised advice were informed whether they had the risk (CT/TT) (*n* = 178) or non-risk (CC) (*n* = 141) alleles of the methylenetetrahydrofolate reductase (*MTHFR*) gene in relation to cardiovascular health and the importance of a sufficient intake of folate. General linear model analysis was used to assess changes in folate intake between the *MTHFR* risk, *MTHFR* non-risk and control groups from baseline to month 6 of the intervention.

**Results:**

There were no differences between the groups for age, gender or BMI. However, there was a significant difference in country distribution between the groups (*p* = 0.010). Baseline folate intakes were 412 ± 172, 391 ± 190 and 410 ± 186 μg per 10 MJ for the risk, non-risk and control groups, respectively. There were no significant differences between the three groups in terms of changes in folate intakes from baseline to month 6. Similarly, there were no changes in reported intake of food groups high in folate.

**Conclusions:**

These results suggest that knowledge of *MTHFR* 677C → T genotype did not improve folate intake in participants with the risk variant compared with those with the non-risk variant.

**Trial registration:**

ClinicalTrials.gov NCT01530139

**Electronic supplementary material:**

The online version of this article (doi:10.1186/s12263-016-0539-x) contains supplementary material, which is available to authorized users.

## Background

The completion of the human genome sequence in the early 2000s promised to revolutionise healthcare through the identification of individuals at increased risk of many complex diseases [1]. Furnished with knowledge of their genotype, it was hypothesised that these individuals would be more likely to make health-promoting changes to ameliorate their risk of disease [2]. To date, a number of studies have investigated the effect of genetic knowledge on changes in lifestyle behaviours in relation to chronic diseases [3–6]. In the REVEAL trial, the investigators reported that in those individuals with a family history of Alzheimer’s disease (AD), knowledge of an *APOE* ɛ4+ risk genotype was positively associated with dietary supplement use [4]. Hendershot and colleagues reported similar effects in relation to alcohol-related cancer risk [7] while others have reported no effect on dietary and lifestyle behaviours in those at risk of breast cancer [8], familial hypercholesterolaemia [9] or diabetes [5].

Following on from this, few studies have examined the impact of genetic knowledge in relation to specific changes in dietary intakes [10, 11]. In relation to the *APOE* genotype, individuals who were told that they had the ɛ4+ risk genotype were found to improve their dietary fat quality more than those individuals with the ɛ4− genotype and control group [10]. Nielsen and El-Sohemy reported a significant reduction in sodium intakes in individuals who were informed they had the risk version of the *ACE* gene compared with those who were given healthy eating advice (*p* = 0.008) [11]. A recent Cochrane review concluded that communicating genotype-based disease risk estimates does not change behaviour in terms of smoking and lifestyle; however, the authors did note a small effect in relation to changes in dietary intake [12, 13]. Therefore, the evidence is mixed and it is still unclear whether knowledge of genotype may promote changes in diet and lifestyle.

This paper examines the impact of methylenetetrahydrofolate reductase (*MTHFR*) genotype disclosure on changes in dietary folate as an example of the potential influence of genomic testing on changes in lifestyle behaviours. *MTHFR* catalyses the conversion of 5,10-methylenetetrahydrofolate to 5-methyltetrahydrofolate, which consequently results in the recycling of homocysteine to methionine in the methylation cycle. The 677C → T polymorphism in *MTHFR* results in three alleles (CC, CT and TT). When compared with normal homozygous variants (677CC), heterozygous (677CT) variants have only 65 % enzyme activity levels and homozygotes (TT) have 30 % enzyme activity [14] which results in decreased circulating folate concentrations; specifically, 10 % lower in heterozygotes and 18 % lower in homozygotes [15].

Because of the lowering effect on plasma homocysteine concentrations, it has been hypothesised that higher intakes of folate and related B vitamins may reduce cardiovascular disease (CVD) risk [16]. Epidemiological studies have predicted that a 3 μmol/L reduction in serum concentration of homocysteine would decrease the risk of coronary heart disease by 11–16 % [17]. However, a recent Cochrane review found no evidence of a positive effect on CVD risk of homocysteine-lowering interventions through supplementation with folate and other B vitamins on CVD [18]. Others have suggested that the beneficial effect of such supplementation will be apparent only in certain population groups with low folate status [19]. Furthermore, supplementation may reduce the risk of stroke rather than other aspects of CVD [20]. Based on these findings, individuals with the risk variants of the *MTHFR* gene (CT and TT) may require higher folate intakes to lower homocysteine concentrations and reduce their CVD risk. The aim of this study was to investigate the influence of knowledge of personal *MTHFR* genotype on changes in folate intake in participants taking part in the Food4Me study (ClinicalTrials.gov number: NCT01530139).

## Methods

### Study design and ethical approval

The Food4Me proof-of-principle (PoP) study was a 6-month Internet-based RCT which aimed to investigate the effect of personalised nutrition advice on health-related outcomes compared with generic healthy eating advice [21]. This study mimicked an online personalised nutrition service where all communication between trained nutritionists and the participants was done via post or electronically and no face-to-face contact took place. Participants were randomised to one of the four treatment groups: level 0 (control group) received non-personalised dietary advice based on standardised European healthy eating guidelines, level 1 received personalised dietary advice based on individual dietary intake data alone, level 2 received personalised dietary advice based on dietary intake and phenotypic markers and level 3 received personalised dietary advice based on dietary intake, phenotypic markers and genotype data. Participants received feedback at 0, 3 and 6 months. A more detailed description of the study protocol has previously been published [21]. Ethical approval was obtained from Research Ethics Committees at the seven participating centres. The study was registered at clinicaltrials.gov (ref NCT01530139). Participants were recruited via the Food4Me website (www.food4me.org) by posters, radio advertisements, leaflets and social media. The recruitment and screening processes have been reported elsewhere [21]. Participants provided informed written consent prior to participation.

### Data collection

All data were self-collected, with participants receiving detailed guidelines on how to collect the data. Data on habitual dietary intake were collected using an on-line food frequency questionnaire (FFQ) which included food items frequently consumed in each of the seven countries. This FFQ was developed and validated [22, 23] specifically for the Food4Me study. To identify possible under-reporting, basal metabolic rate (BMR) was calculated using the Henry equation [24] and multiplied by a PAL factor of 1.1 using the Goldberg cutoffs [25] to determine each individual’s lowest possible estimated energy requirements (EER) when in energy balance. Under-reporting was defined as reported energy intakes below this EER unless participants were following a weight loss diet. Over-reporting was defined as reported energy intakes of greater than 4500 cal [22].

Anthropometric measures included weight (kg), height (m) and waist, hip and thigh circumferences [21]. Buccal cell samples were collected at baseline using a Isohelix SK-1 DNA buccal swab and Isohelix dri-capsule and analysed by LCG Genomics (Hertfordshire, United Kingdom) using KASP™ genotyping assays to provide bi-allelic scoring of single nucleotide polymorphisms (SNPs). *MTHFR* was one of a panel of 33 nutrition-related SNPs measured [21]. Participants were given feedback relating to five key SNPs including *MTHFR*.

### Personalised feedback

Participants randomised to levels 1, 2 and 3 received personalised reports via email, using decision trees for the delivery of systematic tailored dietary advice, from trained nutritionists across seven centres, on dietary, physical activity, phenotypic and genotypic information as appropriate to each group level [21]. These personalised reports were designed based on behaviour change techniques [26, 27]. For example, the first section of the report begun with “a message from your nutritionist” which was a personalised motivating message to encourage the participant to make the relevant dietary and lifestyle changes [28].

Within the report, participants received feedback on their dietary intake and phenotype information using a gradation scale where green indicated “good, no change recommended,” amber indicated “improvement needed” and red indicated “improvement strongly recommended.” Participants in level 3 were informed whether they had a particular “risk” version of the *MTHFR* gene as indicated by “yes” or “no” where risk versions were either of the CT or TT genotypes and non-risk genotype was CC. Participants were informed about the relationship between variants in this gene and their dietary folate needs. With respect to the *MTHFR* risk genotype, participants were informed that “People with a specific variation of this gene can benefit by increasing their intake of the vitamin folate. Increasing folate intake (found in green leafy vegetables) has been associated with an improvement in factors relating to cardiovascular health in these individuals.”

The final section of the report contained a personalised goals section including three individualised nutrient-related goals derived from dietary, phenotypic or genotypic information as appropriate to the intervention group. These nutrient-related goals were selected by a pre-defined nutrient ranking system where nutrients that most warranted change were prioritised [28]. For those in level 3, participants with a *MTHFR* risk genotype and inadequate intakes of folate were advised to increase their folate intake, e.g. “Your total folate intake is below the recommended levels. It is really important for you to increase your folate intake because you have a genetic variation that can benefit by increasing your folate intake.” In contrast, those participants with a *MTHFR* risk genotype and with adequate intakes of folate were given a positive message, i.e. “Your total folate intake is within the recommended levels. You are doing really well because this is a result of your consumption of food rich in folate. We strongly recommend maintaining this level of consumption of foods rich in folate because you have a genetic variation that can benefit from increasing your folate intake. Well done!” As part of the nutrient-related goals message, participants were also given information on the sources of folate rich foods and tips on how to increase their consumption such as eating more dark green leafy vegetables, eating fortified breakfast cereals and adding beans and pulses to salads.

To aid participants’ understanding of genetic risk, additional information was provided to participants on topics such as “what is a genotype” and “how can some genes influence your health status.” This supplementary material was sent in the same email as the personalised reports.

For the purposes of this study, only level 3 participants (who received information about their genotype) and control (level 0) participants are included with changes in dietary intake between 0 and 6 months as the main outcome. For secondary analysis, those participants in level 3 are compared with those in levels 1 and 2 who received personalised advice without genotype information.

### Statistics

Data was analysed using SPSS software version 20 (SPSS Inc. Chicago, Il, USA). Participants were split into *MTHFR* “risk” (CT, TT genotypes) and *MTHFR* “non-risk” (CC genotype) groups and compared with the control group. Following the 6-month intervention, 21 % of individuals who took part in the Food4Me study were lost to follow-up while 8 % dropped out immediately after being randomised. Drop-outs at months 0, 3 and 6 were removed. Descriptive statistics (means and standard deviations) were performed to characterise the groups. Chi-squared analysis was used to investigate categorical variables. ANOVAs were performed to investigate the baseline characteristics. As the data were not normally distributed, variables were log transformed and analysis performed on the log values. General linear models were used to investigate differences between the groups concerning changes in folate intake from baseline to month 6 (and month 3) controlling for baseline folate intakes and country where necessary.

To address the research question “Does knowledge of *MTHFR* genotype improve folate intake more in those with risk version of the gene compared with those with the non-risk version of the gene and those who received general healthy eating advice (i.e. control group),” the following analysis was conducted: (1) the change in folate intake from month 0 to month 6 for all participants in the *MTHFR* risk, *MTHFR* non-risk and control groups; (2) the change in folate intake from month 0 to month 6 between the control group and those in the *MTHFR* risk and *MTHFR* non-risk groups restricted to those who received a folate-related goal (i.e. those who were told that they needed to increase their folate intake and those who were told to maintain their current folate intakes) and (3) the change in folate intake from month 0 to month 6 between the control group and those in the *MTHFR* risk and *MTHFR* non-risk groups restricted to those who were told that they needed to increase their folate. Fisher’s least significant difference (LSD) post hoc analysis was used to investigate inter-group differences. As secondary analysis, it was also investigated whether personalised advice based on *MTHFR* risk knowledge was more effective in motivating changes in dietary folate compared with those who received personalised advice with no *MTHFR* genotype information. To examine the effect of personalisation of dietary advice on changes in dietary folate, differences between the control group, level 1 group and level 2 group were also assessed. All analyses were repeated using data for valid reporters only, i.e. after removal of under-reporters and over-reporters as defined previously.

## Results

### Baseline characteristics of the *MTHFR* risk (CT/TT), *MTHFR* non-risk (CC) and control groups

There were no differences between the risk, non-risk and control groups in terms of age and anthropometric measures (Table 1). All groups had a BMI that was slightly above the normal BMI range (25.5 ± 4.9, 26.0 ± 4.9 and 25.1 ± 4.4 kg/m^2^, respectively), and there was no difference in gender distribution between the groups. Distribution of the risk and non-risk groups were significantly different across the countries (*p* = 0.010) (Table 1). The frequency of the *MTHFR* risk variant was the highest in Germany and the lowest in Poland. Overall, the genotype frequencies (CC, CT, TT) were within the Hardy Weinburg Equilibrium (results not shown). Intakes of energy, folate and major folate-containing foods for each of the three groups at month 0 and month 6 are given in Table 2. At baseline, folate intakes for the risk, non-risk and control groups were similar at 412 ± 172, 391 ± 190 and 410 ± 186 μg per 10 MJ of energy, respectively. One outlier was removed from the analysis due to consumption of a medically prescribed high folate supplement (>5000 mcg) between months 3 and 6.

**Table 1:** Baseline characteristics of *MTHFR* risk, *MTHFR* non-risk and control groups

Demographical information	*MTHFR* Risk (CT/TT) (*n* = 178)	*MTHFR* Non-risk (CC) (*n* = 141)	Control (*n* = 309)	*p* value^a^
Age (years)	42 ± 13	41 ± 14	40 ± 13	0.526
Gender (M/F)	71/107	68/73	130/179	0.304
Weight (kg)	75.1 ± 15.4	76.5 ± 16.1	73.77 ± 15.02	0.216
BMI (kg/m^2^)	25.5 ± 4.9	26.0 ± 5.0	25.1 ± 4.4	0.138
W.C. (m)	0.86 ± 0.13	0.88 ± 0.14	0.85 ± 0.13	0.129
Frequency % (*n*)^b^				
Germany	19.1 (34)	7.8 (11)		0.010
Greece	17.4 (31)	13.5 (19)		
Ireland	10.7 (19)	17.1 (24)		
Netherlands	16.3 (29)	17.7 (25)		
Poland	8.4 (15)	15.6 (22)		
Spain	16.3 (29)	11.3 (16)		
UK	11.8 (21)	17.0 (24)		

Excludes drop-outs at months 3 and 6

*W.C.* waist circumference

^a^Baseline characteristics presented as means ± standard deviations and differences between the groups were investigated using ANOVA for all variables with the exception of gender and country where chi-square analysis was used

^b^Frequency of country was assessed across the *MTHFR* risk and *MTHFR* non-risk groups only

**Table 2:** Comparison of dietary intakes for the *MTHFR* risk, *MTHFR* non-risk and control groups at M0 and M6

	*MTHFR* risk (CT/TT)	Number	*MTHFR* non-risk (CC)	Number	Control	Number	*p* value^a^
Energy (kJ) M0	10,201 ± 3423	178	11,558 ± 5479	141	10,617 ± 4810	309	0.203
M6	8810 ± 2968	178	9637 ± 3675	141	9605 ± 4132	309	
Folate (μg per 10 MJ) M0	412 ± 172	178	391 ± 190	141	410 ± 186	309	0.131
M6	427 ± 193	178	410 ± 168	141	410 ± 210	309	
Liver (g) M0	1 ± 3	178	2 ± 9	141	1 ± 3	309	0.162
M6	1 ± 4	178	1 ± 4	141	1 ± 3	309	
Poultry (g) M0	33 ± 42	144	37 ± 40	130	30 ± 29	270	0.136
M6	33 ± 32	144	32 ± 28	130	32 ± 52	270	
Shellfish (g) M0	4 ± 7	178	3 ± 7	141	3 ± 5	309	0.430
M6	4 ± 7	178	3 ± 6	141	3 ± 7	309	
Green leafy veg (g) M0	49 ± 41	178	48 ± 44	141	45 ± 42	309	0.220
M6	53 ± 50	178	49 ± 45	141	46 ± 48	309	
Fortified cereals (g) M0	22 ± 33	178	20 ± 31	141	19 ± 27	309	0.444
M6	22 ± 30	178	20 ± 24	141	20 ± 26	309	
Beans and legumes (g) M0	22 ± 34	144	27 ± 34	130	25 ± 44	270	0.726
M6	20 ± 23	144	26 ± 48	130	21 ± 31	269	

Excludes drop-outs at months 3 and 6

*M0* month 0, *M6* month 6

^a^Values presented as means ± standard deviations. All analysis was conducted on log-transformed values. General linear models were used to assess the impact of group on month 6 intake with M0 as a covariate and controlling for country where necessary

### Changes in dietary folate intakes from baseline to month 6

Dietary folate intakes increased from 412 ± 172 μg per 10 MJ to 427 ± 193 μg per 10 MJ in the *MTHFR* risk group and from 391 ± 190 μg per 10 MJ to 410 ± 168 μg per 10 MJ in the *MTHFR* non-risk whereas no increase was observed in the control group. Although both intervention groups (risk and non-risk) increased their folate intakes in comparison with the control, there were no significant differences between the groups (*p* = 0.131). There were no significant differences between the risk, non-risk or control with respect to changes in reported intakes of food groups high in folate (Table 2). Similarly, there were no differences in frequency of folate supplement users between the groups at any of the time points (data not shown). Table 3 illustrates the dietary intakes of those individuals who received a folate-related goal (i.e. those who were advised to increase their folate intake and those who were advised to maintain their current folate intakes) compared with the control group. Post hoc analysis revealed a significant (*p* = 0.033) difference between the non-risk and control groups for change in folate intake from baseline. No significant differences were observed between the groups with respect to changes in intakes of food groups containing folate. Table 4 summarises the folate intakes of those individuals who were advised specifically to increase their folate intake compared with the control group. There were no significant differences between the groups for changes in folate intakes or of folate-containing food groups.

**Table 3:** Dietary intakes by *MTHFR* risk and *MTHFR* non-risk participants who received folate-related goal (i.e. those who were told that they needed to increase their folate intake and those who were told to maintain their current folate intakes) compared with those who received generic healthy eating advice (control group)

	MTHFR risk (CT/TT)	Number	MTHFR non-risk (CC)	Number	Control	Number	*p* value^a^
Energy (kJ) M0	9506 ± 3225	121	9013 ± 2873	57	10,617 ± 4810	309	0.325
M6	8589 ± 3028	121	7859 ± 2199	57	9605 ± 4132	309	
Folate (μg per 10 MJ) M0	402 ± 156	121	330 ± 80	57	410 ± 186	309	0.033
M6	429 ± 198	121	398 ± 172^c^	57	410 ± 210^n^	309	
Liver (g) M0	1 ± 3	121	2 ± 4	57	1 ± 3	309	0.074
M6	2 ± 4	121	1 ± 4	57	1 ± 3	309	
Poultry (g) M0	34 ± 47	100	27 ± 29	52	30 ± 29	270	0.096
M6	34 ± 34	100	24 ± 25	52	32 ± 52	270	
Shellfish (g) M0	4 ± 7	121	3 ± 6	57	3 ± 5	309	0.167
M6	3 ± 5	121	2 ± 5	57	3 ± 7	309	
Green leafy veg (g) M0	46 ± 43	121	42 ± 43	57	45 ± 42	309	0.208
M6	49 ± 38	121	44 ± 36	57	46 ± 48	309	
Fortified cereals (g) M0	23 ± 37	121	11 ± 15	57	19 ± 27	309	0.304
M6	24 ± 32	121	14 ± 16	57	20 ± 26	308	
Beans and legumes (g) M0	22 ± 35	100	20 ± 24	52	25 ± 44	270	0.443
M6	22 ± 24	100	27 ± 63	52	21 ± 31	269	

Includes participants who received folate as a target nutrient at month 0 and/or month 3 and excludes drop-outs at months 3 and 6

*M0* month 0, *M6* month 6

^a^Values are presented as means ± standard deviations. All analysis was conducted on the log transformed values. General linear models were used to assess the impact of group on month 6 intake with M0 as a covariate and controlling for country where necessary. Superscript letters denote where the differences lie between groups where superscript letter *n* means significantly different from the *MTHFR* non-risk group and superscript letter *c* means significantly different from the control group

**Table 4:** Dietary intakes by *MTHFR* risk and *MTHFR* non-risk participants who were told to increase their folate intake compared with those who received general healthy eating advice (control group)

	*MTHFR* risk (CT/TT)	Number	*MTHFR* non-risk (CC)	Number	Control	Number	*p* value^a^
Energy (kJ) M0	8620 ± 2708	83	8939 ± 2879	55	10,617 ± 4810	309	0.061
M6	7767 ± 2742	83	7842 ± 2237	55	9605 ± 4132	309	
Folate (μg per 10 MJ) M0	361 ± 123	83	329 ± 81	55	410 ± 186	309	0.165
M6	385 ± 147	83	395 ± 175	55	410 ± 210	309	
Liver (g) M0	1 ± 3	83	2 ± 4	55	1 ± 3	309	0.369
M6	2 ± 4	83	1 ± 4	55	1 ± 3	309	
Poultry (g) M0	36 ± 53	66	27 ± 30	50	30 ± 29	270	0.072
M6	35 ± 28	66	25 ± 25	50	32 ± 55	270	
Shellfish (g) M0	3 ± 6	83	3 ± 6	55	3 ± 5	309	0.092
M6	2 ± 3	83	2 ± 5	55	3 ± 7	309	
Green leafy veg (g) M0	39 ± 38	83	38 ± 40	55	45 ± 42	309	0.261
M6	44 ± 33	83	42 ± 35	55	46 ± 48	309	
Fortified cereals (g) M0	14 ± 19	83	11 ± 16	55	19 ± 27	309	0.469
M6	17 ± 21	83	14 ± 16	55	20 ± 26	308	
Beans and legumes (g) M0	17 ± 17	66	19 ± 24	50	25 ± 44	270	0.146
M6	22 ± 22	66	27 ± 64	50	21 ± 31	269	

Includes participants who were specifically advised to increase their folate intakes at month 0 and/or month 3 and where drop-outs at months 3 and 6 were excluded

*M0* month 0, *M6* month 6

^a^Values are presented as means ± standard deviations. All analysis was conducted on the log-transformed values. General linear models were used to assess the impact of group on month 6 with M0 intake as a covariate and controlling for country where necessary

Given the significant difference between the groups in terms of the *MTHFR* genotype frequency, the change in folate intakes was also investigated per country and no significant differences were found. Changes in dietary folate intakes between month 0 and month 3 were also investigated (Additional file 1: Table S1, S2 and S3). Overall, no significant differences were found between the groups. No differences were also found when those in the *MTHFR* risk and *MTHFR* non-risk groups were compared with those who received personalised advice without information on *MTHFR* genotype (Additional file 1: Table S4 and S5). No differences were observed between the control, level 1 and level 2 groups in terms of changes in dietary folate intakes from baseline to month 3 or month 6 (data not shown). All of the analyses were repeated for “valid” dietary reporters (i.e. after exclusion of both over- and under-reporters), and similar results were observed (data not shown).

## Discussion

This study demonstrated that knowledge of carriage of the risk variant (CT, TT) for the *MTHFR* 677C → T genotype did not improve folate intake compared with participants with the non-risk variant (CC) in the Food4Me study. These findings add to the current literature regarding disclosure of genotype-based advice.

The evidence supporting the benefits of genetic risk knowledge is mixed, with some studies demonstrating a benefit of genetic knowledge in motivating lifestyle changes [4, 11, 29, 30] and others reporting no significant effect [5, 31, 32]. The majority of such studies investigated the effect of knowledge of genotype-based risk on motivation to change lifestyle including diet with respect to one specific disease, e.g. diabetes [5] or CVD [3]. Grant and colleagues investigated the effect of genetic risk testing and counselling on motivation to change behaviours for the reduction of diabetes risk [5]. In this trial, overweight patients at increased phenotypic risk of diabetes were randomised to receive genetic testing or not receive genetic testing, and then, both groups participated in a 12-week diabetes prevention programme. The investigators found that the genetic risk counselling did not alter significantly self-reported motivation or adherence to the prevention programme [5]. Taylor and colleagues examined lifestyle changes among urban African-American women following genetic counselling for hypertension compared to baseline [3]. With the exception of sodium intake, changes in lifestyle behaviours, blood pressure and pulse pressure readings did not differ significantly from baseline [3]. The hypothesis that communicating risk of developing Crohn’s disease based on genotype can motivate behaviour change among smokers at familial risk was also investigated [33]. The researchers found that the addition of genotypic information when communicating risk for Crohn’s disease based on family history and smoking status did not affect motivation for behaviour change [33]. The results of the present study are in line with these findings of a lack of effect of knowledge of genotype-based risk.

However, in contrast, a recent study undertaken with young adults in Canada observed a positive effect of disclosing genetic information on changes in diet [11]. In this study, participants (*n* = 157) were genotyped for variants that affect caffeine metabolism (*CYP1A2*), vitamin C utilisation (*GSTT1* and *GSTM1*), sweet taste perception (*TAS1R2*) and sodium-sensitivity (*ACE*). They were then randomised to receive either personalised nutrition advice based on individual genotype or generic healthy eating guidance. After 3 months, there were no significant dietary changes between the intervention and control groups, but at 12 months, participants with the risk version of the *ACE* gene in the intervention group significantly reduced their sodium intake compared with the control group (*p* = 0.008). These findings may mean that a longer time frame is needed to observe the added benefit from genotype-based advice. In the Canadian study, there were no significant changes for dietary targets other than salt perhaps because the participants were already consuming intakes of those dietary components in line with the recommendations [11].

The current study was part of the larger Food4Me study which investigated the effect of varying levels of personalised advice on motivating behaviour change compared with generic healthy eating advice [21]. It should be noted that this study was not designed to examine the effect of disclosure of *MTHFR* genotype specifically and related dietary changes to folate. Since each participant randomised to the personalised nutrition group (levels 1–3) received three individualised dietary goals, it is possible that participants could have prioritised other aspects of their personalised dietary advice so the impact of the *MTHFR*-related advice was diminished. It is also likely that participants were more interested in weight loss and healthy eating advice as approximately 50 % of the participants were overweight or obese. Furthermore, dietary advice to reduce saturated fat and salt intake and related health benefits would be better known to participants from a public health point of view in comparison with dietary advice to increase folate to reduce CVD risk. This study was designed to mimic an online personalised nutrition company where tailored dietary advice was delivered via email by trained nutritionists. Although additional information was provided to participants regarding genetic risk and related dietary intake, it is possible that the online delivery of the information may have affected participants’ understanding of their genetic results and personalised dietary information which could have contributed to the unchanged dietary behaviour observed. In addition, as noted above, a longer time frame may be needed to reveal any additional effect of genotype-based advice [11].

The volunteers in the Food4Me study were recruited on the basis that they were generally healthy. This is in contrast to some other studies which have focused on particular patient groups or those at increased phenotypic or familial disease risk. In the REVEAL trial, the investigators examined the effect of disclosure of *APOE* genotype-based risk of AD on related lifestyle changes [30]. The investigators reported that those with the *APOE4*-positive genotype were significantly more likely to report making an AD-specific health behaviour change 1 year after disclosure compared to those who were *APOE4*-negative (*p* = 0.02). In a follow-up study, Vernarelli and colleagues reported that *APOE4*-positive individuals with family history of AD were twice as likely to report making a nutrition behaviour change than those who were *APOE4* negative with an increase in supplement use among *APOE4*-positive participants [4]. However, in critique of the REVEAL study, Fanshawe and colleagues drew attention to the fact that those who were *APOE4* positive also had a higher AD-risk score based on family history so that the greater behaviour may have been a consequence of information about higher risk estimate and that genotypic information was not the key motivator for behaviour change [34]. Whether individuals at increased risk of a particular disease are more responsive to genotype-based dietary advice per se remains an open question.

Strengths of this study include the randomised design and the fact that it mimicked a personalised nutrition service similar to those currently available. The main limitation of the study is the use of a FFQ to quantify the changes in intakes of dietary folate. While FFQs are useful for examining population level intakes, they are less good at examining individual dietary intakes and potentially, measures of circulating folate concentrations may be more sensitive in capturing changes in folate intake [35]. Furthermore, the Food4Me study was not designed to examine changes in specific nutrients as participants were given a selection of nutrients to change, and therefore, it would be challenging to identify changes in any one particular nutrient. The population studied could also have been a limitation as the personalised nutrition advice was given to individuals free of charge, and those who pay for such services may be more motivated to make the relevant dietary and lifestyle changes.

## Conclusions

In summary, the findings of this study suggest that knowledge of *MTHFR* variant status did not influence changes in dietary folate intake in response to a personalised nutrition intervention. Our findings are similar to those studies which showed no effect of genotypic information on relevant dietary and lifestyle changes [5, 33]. Future work should be directed towards testing this hypothesis in individuals at a known higher phenotypic or familial risk of CVD and should include the measurement of blood-based markers of folate status. Furthermore, it would be interesting to test this concept in a general practitioner (GP) setting where face-to-face contact between the individual and healthcare provider may result in a different outcome compared with online delivery of personalised nutrition and lifestyle advice.

## Abbreviations

AD, Alzheimer’s disease; BMR, basal metabolic rate; CVD, cardiovascular disease; EER, estimated energy requirement; FFQ, food frequency questionnaire; GP, general practitioner; LSD, least significant difference; MTHFR, methylenetetrahydrofolate reductase; PoP, proof-of-principle study; SNPs, single nucleotide polymorphisms

## Additional file

Additional file 1: Table S1. Dietary intakes for the *MTHFR* risk, *MTHFR* non-risk and control groups at M0 and M3. **Table S2.** Dietary intakes by *MTHFR* risk and *MTHFR* non-risk participants who received folate-related goal (i.e. those who were told to increase their folate intake and those who were told to maintain their current folate intakes) compared with those who received generic healthy eating advice at M0 and M3. **Table S3.** Dietary intakes by *MTHFR* risk and *MTHFR* non-risk participants who were told to increase their folate intake compared with those who received generic healthy eating advice at M0 and M3. **Table S4.** Comparison of dietary intakes for the *MTHFR* risk, *MTHFR* non-risk and those who received personalised nutrition advice without genotype (levels 1 and 2) at M0 and M6. **Table S5.** Comparison of dietary intakes for the *MTHFR* risk, *MTHFR* non-risk and those who received personalised nutrition advice without genotype (levels 1 and 2) at M0 and M3. (DOCX 36 kb)
