# Supplementary material for: The impact of MTHFR 677C → T risk knowledge on changes in folate intake: findings from the Food4Me study
Source: Genes Nutr. 2016 Sep 29;11:25. doi: 10.1186/s12263-016-0539-x (PMC5043523; doi:10.1186/s12263-016-0539-x)
Supplement: Additional file 1: Table S1. — Dietary intakes for the MTHFR risk, MTHFR non-risk and control groups at M0 and M3. Table S2. Dietary intakes by MTHFR risk and MTHFR non-risk participants who received folate-related goal (i.e. those who were told to increase their folate intake and those who were told to maintain their current folate intakes) compared with those who received generic healthy eating advice at M0 and M3. Table S3. Dietary intakes by MTHFR risk and MTHFR non-risk participants who were told to increase their folate intake compared with those who received generic healthy eating advice at M0 and M3. Table S4. Comparison of dietary intakes for the MTHFR risk, MTHFR non-risk and those who received personalised nutrition advice without genotype (levels 1 and 2) at M0 and M6. Table S5. Comparison of dietary intakes for the MTHFR risk, MTHFR non-risk and those who received personalised nutrition advice without genotype (levels 1 and 2) at M0 and M3. (DOCX 36 kb) [file 12263_2016_539_MOESM1_ESM.docx]

**Additional files**

**Article title -** The impact of *MTHFR* 677C→T risk knowledge on changes in folate intake: findings from the Food4Me study

**Journal name –** Genes and Nutrition

**Author names -** Clare B O’Donovan, Marianne C Walsh, Hannah Forster, Clara Woolhead, Carlos Celis-Morales, Rosalind Fallaize, Anna L Macready, Cyril F M Marsaux, Santiago Navas-Carretero, Rodrigo San-Cristobal, Silvia Kolossa, Christina Mvrogianni, Christina P Lambrinou, George Moschonis, Magdalena Godlewska, Agnieszka Surwillo, Jildau Bouwman, Keith Grimaldi, Iwona Traczyk, Christian A Drevon, Hannelore Daniel, Yannis Manios, J Alfredo Martinez, Wim H M Saris, Julie A Lovegrove, John C Mathers, Michael J Gibney, Lorraine Brennan and Eileen R Gibney

**Affiliation and e-mail address of the corresponding author -** Dr. Eileen R Gibney, Institute of Food & Health, University College Dublin, Dublin 4, Ireland. Email – [eileen.gibney@ucd.ie](mailto:eileen.gibney@ucd.ie). Telephone – (01) 716 2819.

**Table S1** Dietary intakes for the *MTHFR* risk, *MTHFR* non-risk and control groups at M0 and M3^a^

|  | *MTHFR* Risk (CT/TT) | n | *MTHFR* Non-Risk  (CC) | n | Control | n | p value^b^ |
| --- | --- | --- | --- | --- | --- | --- | --- |
| *Energy (kJ)* M0 | 10201 ± 3423 | 178 | 11558 ± 5479 | 141 | 10617 ± 4810 | 309 | 0.028 |
| M3 | 9050 ± 2808^c^ | 178 | 9485 ± 3494^c^ | 141 | 9898 ± 3856^r,n^ | 309 |  |
| *Folate (µg per 10MJ)* M0 | 412 ± 172 | 178 | 391 ± 190 | 141 | 410 ± 186 | 309 | 0.325 |
| M3 | 433 ± 226 | 178 | 408 ± 168 | 141 | 414 ± 184 | 309 |  |
| *Liver (g)* M0 | 1 ± 3 | 178 | 2 ± 9 | 141 | 1 ± 3 | 309 | 0.845 |
| M3 | 1 ± 2 | 178 | 1 ± 3 | 141 | 1 ± 4 | 309 |  |
| *Poultry (g*) M0 | 33 ± 42 | 144 | 37 ± 40 | 130 | 30 ± 29 | 270 | 0.268 |
| M3 | 32 ± 40 | 144 | 36 ± 44 | 130 | 29 ± 36 | 270 |  |
| *Shellfish (g)* M0 | 4 ± 7 | 178 | 3 ± 7 | 141 | 3 ± 5 | 309 | 0.204 |
| M3 | 4 ± 7 | 178 | 3 ± 9 | 141 | 4 ± 8 | 309 |  |
| *Green leafy veg* (g) M0 | 49 ± 41 | 178 | 48 ± 44 | 141 | 45 ± 42 | 309 | 0.862 |
| M3 | 47 ± 37 | 178 | 47 ± 43 | 141 | 47 ± 45 | 309 |  |
| *Fortified cereals (g)* M0 | 22 ± 33 | 178 | 20 ± 31 | 141 | 19 ± 27 | 309 | 0.263 |
| M3 | 23 ± 30 | 178 | 23 ± 31 | 141 | 21 ± 28 | 309 |  |
| *Beans & legumes (g)* M0 | 22 ± 34 | 144 | 27 ± 34 | 130 | 25 ± 44 | 270 | 0.494 |
| M3 | 18 ± 23 | 178 | 23 ± 32 | 130 | 19 ± 24 | 270 |  |

^a^Excludes drop-outs at months 3 and 6.

^b^Values presented as means ± standard deviations. All analysis was conducted on the log transformed values. General linear models were used to assess the impact of group on month 3 intake with M0 intake as a covariate and controlling for country where necessary. Superscript letters denote where the differences lie between groups where ^r^ means significantly different from the *MTHFR* risk group, ^n^ means significantly different from the *MTHFR* non-risk group and ^c^ means significantly difference from the control group.

M0, month 0; M3, month 3.

**Table S2** Dietary intakes by *MTHFR* risk and *MTHFR* non-risk participants who received folate-related goal (i.e. those who were told to increase their folate intake and those who were told to maintain their current folate intakes) compared with those who received generic healthy eating advice at M0 and M3^a^

|  | *MTHFR* Risk  (CT/TT) | n | *MTHFR* Non-Risk  (CC) | n | Control | n | p value^b^ |
| --- | --- | --- | --- | --- | --- | --- | --- |
| *Energy (kJ)* M0 | 9506 ± 3225 | 121 | 9013 ± 2873 | 57 | 10617 ± 4810 | 309 | <0.001 |
| M3 | 8589 ± 3028^c^ | 121 | 7697 ± 2977^c^ | 57 | 9898 ± 3856^r,n^ | 309 |  |
| *Folate ( µg per 10MJ)* M0 | 402 ± 156 | 121 | 330 ± 80 | 57 | 410 ± 186 | 309 | 0.314 |
| M3 | 431 ± 233 | 121 | 372 ± 164 | 57 | 414 ± 185 | 309 |  |
| *Liver (g)* M0 | 1 ± 3 | 121 | 2 ± 4 | 57 | 1 ± 3 | 309 | 0.601 |
| M3 | 1 ± 2 | 121 | 1 ± 2 | 57 | 1 ± 4 | 309 |  |
| *Poultry (g*) M0 | 34 ± 47 | 100 | 27 ± 29 | 52 | 30 ± 29 | 270 | 0.273 |
| M3 | 32 ± 45 | 100 | 33 ± 59 | 52 | 29 ± 36 | 270 |  |
| *Shellfish (g)* M0 | 4 ± 7 | 121 | 3 ± 6 | 57 | 3 ± 5 | 309 | 0.041 |
| M3 | 3 ± 6 | 121 | 2 ± 3^c^ | 57 | 4 ± 8^n^ | 308 |  |
| *Green leafy veg* (g) M0 | 46 ± 43 | 121 | 42 ± 43 | 57 | 45 ± 42 | 309 | 0.741 |
| M3 | 43 ± 37 | 121 | 38 ± 32 | 57 | 47 ± 45 | 309 |  |
| *Fortified cereals (g)* M0 | 23 ± 37 | 121 | 11 ± 15 | 57 | 19 ± 27 | 309 | 0.234 |
| M3 | 24 ± 33 | 121 | 16 ± 28 | 57 | 21 ± 28 | 309 |  |
| *Beans & legumes (g)* M0 | 22 ± 35 | 100 | 20 ± 24 | 52 | 25 ± 44 | 270 | 0.800 |
| M3 | 17 ± 21 | 100 | 16 ± 18 | 52 | 19 ± 24 | 270 |  |

^a^Includes participants who received folate as a target nutrient at month 0 and/or month 3 and where drop-outs at months 3 and 6 were excluded.

^b^Values are presented as means ± standard deviations. All analysis was conducted on the log transformed values. General linear models were used to assess the impact of group on month 3 intake with M0 intake as a covariate and controlling for country where necessary. Superscript letters denote where the differences lie between groups where ^r^ means significantly different from the *MTHFR* risk group, ^n^ means significantly different from the *MTHFR* non-risk group and ^c^ means significantly difference from the control group.

M0, month 0; M3, month 3.

**Table S3** Dietary intakes by *MTHFR* risk and *MTHFR* non-risk participants who were told to increase their folate intake compared with those who received generic healthy eating advice at M0 and M3^a^

|  | *MTHFR* Risk  (CT/TT) | n | *MTHFR* Non-Risk (CC) | n | Control | n | p value^b^ |
| --- | --- | --- | --- | --- | --- | --- | --- |
| *Energy (kJ)* M0 | 8620 ± 2708 | 83 | 8939 ± 2879 | 55 | 10617 ± 4810 | 309 | <0.001 |
| M3 | 7546 ± 2261^c^ | 83 | 7459 ± 2214^c^ | 55 | 9898 ± 3856^r,n^ | 309 |  |
| *Folate (µg per 10MJ)* M0 | 361 ± 123 | 83 | 329 ± 81 | 55 | 410 ± 186 | 309 | 0.338 |
| M3 | 383 ± 170 | 83 | 362 ± 157 | 55 | 414 ± 184 | 309 |  |
| *Liver (g)* M0 | 1 ± 3 | 83 | 2 ± 4 | 55 | 1 ± 3 | 309 | 0.214 |
| M3 | 1 ± 3 | 83 | 1 ± 2 | 55 | 1 ± 4 | 309 |  |
| *Poultry (g*) M0 | 36 ± 53 | 66 | 27 ± 30 | 50 | 30 ± 29 | 270 | 0.322 |
| M3 | 29 ± 24 | 66 | 33 ± 60 | 50 | 29 ± 36 | 270 |  |
| *Shellfish (g)* M0 | 3 ± 6 | 83 | 3 ± 6 | 55 | 3 ± 5 | 309 | 0.050 |
| M3 | 3 ± 4 | 83 | 2 ± 4^c^ | 55 | 4 ± 8^n^ | 308 |  |
| *Green leafy veg* (g) M0 | 39 ± 38 | 83 | 38 ± 40 | 55 | 45 ± 42 | 309 | 0.900 |
| M3 | 37 ± 29 | 83 | 37 ± 31 | 55 | 47 ± 45 | 309 |  |
| *Fortified cereals (g)* M0 | 14 ± 19 | 83 | 11 ± 16 | 55 | 19 ± 27 | 309 | 0.824 |
| M3 | 15 ± 17 | 83 | 14 ± 22 | 55 | 21 ± 28 | 309 |  |
| *Beans & legumes (g)* M0 | 17 ± 17 | 66 | 19 ± 24 | 50 | 25 ± 44 | 270 | 0.541 |
| M3 | 13 ± 16 | 66 | 15 ± 16 | 50 | 19 ± 24 | 270 |  |

^a^Includes participants who were specifically advised to increase their folate intakes at month 0 and/or month 3 and where drop-outs at months 3 and 6 were excluded.

^b^Values are presented as means ± standard deviations. All analysis was conducted on the log transformed values. General linear models were used to assess the impact of group on month 3 intake with M0 intake as a covariate and controlling for country where necessary. Superscript letters denote where the differences lie between groups where ^r^ means significantly different from the *MTHFR* risk group, ^n^ means significantly different from the *MTHFR* non-risk group and ^c^ means significantly difference from the control group.

M0, month 0; M3, month 3.

**Table S4** Comparison of dietary intakes for the *MTHFR* risk, *MTHFR* non-risk and those who received personalised nutrition advice without genotype (Levels 1 and 2) at M0 and M6^a^

|  | *MTHFR* Risk  (CT/TT) | n | *MTHFR* Non-Risk  (CC) | n | Levels 1 and 2 | n | p value^b^ |
| --- | --- | --- | --- | --- | --- | --- | --- |
| *Energy (kJ)* M0 | 10201 ± 3422 | 178 | 11558 ± 5479 | 141 | 10712 ± 4327 | 634 | 0.826 |
| M6 | 8810 ± 2968 | 178 | 9637 ± 3675 | 141 | 9264 ± 3730 | 634 |  |
| *Folate (µg per 10MJ)* M0 | 412 ± 172 | 178 | 391 ± 190 | 141 | 400 ± 186 | 634 | 0.644 |
| M6 | 427 ± 193 | 178 | 410 ± 168 | 141 | 410 ± 163 | 634 |  |
| *Liver (g)* M0 | 1 ± 3 | 178 | 2 ± 9 | 141 | 2 ± 4 | 634 | 0.557 |
| M6 | 1 ± 4 | 178 | 1 ± 4 | 141 | 1 ± 3 | 634 |  |
| *Poultry (g*) M0 | 33 ± 42 | 144 | 37 ± 40 | 130 | 32 ± 31 | 545 | 0.466 |
| M6 | 33 ± 32 | 144 | 32 ± 28 | 130 | 31 ± 27 | 545 |  |
| *Shellfish (g)* M0 | 4 ± 7 | 178 | 3 ± 7 | 141 | 3 ± 5 | 634 | 0.215 |
| M6 | 4 ± 7 | 178 | 3 ± 6 | 141 | 3 ± 5 | 634 |  |
| *Green leafy veg* (g) M0 | 49 ± 41 | 178 | 48 ± 44 | 141 | 44 ± 40 | 634 | 0.079 |
| M6 | 53 ± 50 | 178 | 49 ± 45 | 141 | 44 ± 40 | 634 |  |
| *Fortified cereals (g)* M0 | 22 ± 33 | 178 | 20 ± 31 | 141 | 19 ± 26 | 634 | 0.838 |
| M6 | 22 ± 30 | 178 | 20 ± 24 | 141 | 21 ± 30 | 634 |  |
| *Beans & legumes (g)* M0 | 22 ± 34 | 144 | 27 ± 34 | 130 | 21 ± 28 | 545 | 0.686 |
| M6 | 20 ± 23 | 144 | 26 ± 48 | 130 | 19 ± 23 | 545 |  |

^a^Excludes drop-outs at months 3 and 6.

^b^Values presented as means and standard deviations. All analysis was conducted on log transformed values. General linear models were used to assess the impact of group on month 6 intake with M0 as a covariate and controlling for country where necessary.

M0, month 0; M6, month 6.

**Table S5** Comparison of dietary intakes for the *MTHFR* risk, *MTHFR* non-risk and those who received personalised nutrition advice without genotype (Levels 1 and 2) at M0 and M3^a^

|  | *MTHFR* Risk  (CT/TT) | n | *MTHFR* Non-Risk  (CC) | n | Levels 1 and 2 | n | p value^b^ |
| --- | --- | --- | --- | --- | --- | --- | --- |
| *Energy (kJ)* M0 | 10201 ± 3422 | 178 | 11558 ± 5479 | 141 | 10712 ± 4327 | 634 | 0.597 |
| M3 | 9050 ± 2968 | 178 | 9485 ± 3494 | 141 | 9186 ± 3560 | 634 |  |
| *Folate (µg per 10MJ)* M0 | 412 ± 172 | 178 | 391 ± 190 | 141 | 400 ± 186 | 634 | 0.411 |
| M3 | 433 ± 226 | 178 | 407 ± 168 | 141 | 421 ± 187 | 634 |  |
| *Liver (g)* M0 | 1 ± 3 | 178 | 2 ± 9 | 141 | 2 ± 4 | 634 | 0.599 |
| M3 | 1 ± 2 | 178 | 1 ± 3 | 141 | 2 ± 4 | 634 |  |
| *Poultry (g*) M0 | 33 ± 42 | 144 | 37 ± 40 | 130 | 32 ± 31 | 545 | 0.598 |
| M3 | 32 ± 40 | 144 | 36 ± 44 | 130 | 31 ± 31 | 545 |  |
| *Shellfish (g)* M0 | 4 ± 7 | 178 | 3 ± 7 | 141 | 3 ± 5 | 634 | 0.810 |
| M3 | 4 ± 7 | 178 | 3 ± 9 | 141 | 3 ± 5 | 634 |  |
| *Green leafy veg* (g) M0 | 49 ± 41 | 178 | 48 ± 44 | 141 | 44 ± 40 | 634 | 0.643 |
| M3 | 44 ± 35 | 178 | 45 ± 41 | 141 | 41 ± 39 | 634 |  |
| *Fortified cereals (g)* M0 | 22 ± 33 | 178 | 20 ± 31 | 141 | 19 ± 26 | 634 | 0.897 |
| M3 | 23 ± 30 | 178 | 23 ± 31 | 141 | 20 ± 25 | 634 |  |
| *Beans & legumes (g)* M0 | 22 ± 34 | 144 | 27 ± 34 | 130 | 21 ± 28 | 545 | 0.463 |
| M3 | 18 ± 23 | 144 | 23 ± 32 | 130 | 18 ± 26 | 545 |  |

^a^Excludes drop-outs at months 3 and 6.

^b^Values presented as means and standard deviations. All analysis was conducted on log transformed values. General linear models were used to assess the impact of group on month 3 intake with M0 as a covariate and controlling for country where necessary.

M0, month 0; M3, month 3.
